# Supplementary material for: Systematic Analysis and Identification of Drought-Responsive Genes of the CAMTA Gene Family in Wheat (Triticum aestivum L.)
Source: Int J Mol Sci. 2022 Apr 20;23(9):4542. doi: 10.3390/ijms23094542 (PMC9102227; doi:10.3390/ijms23094542)
Supplement: Supplementary file 1 [file ijms-23-04542-s001.zip › CAMTA Supplement Figures.pdf]

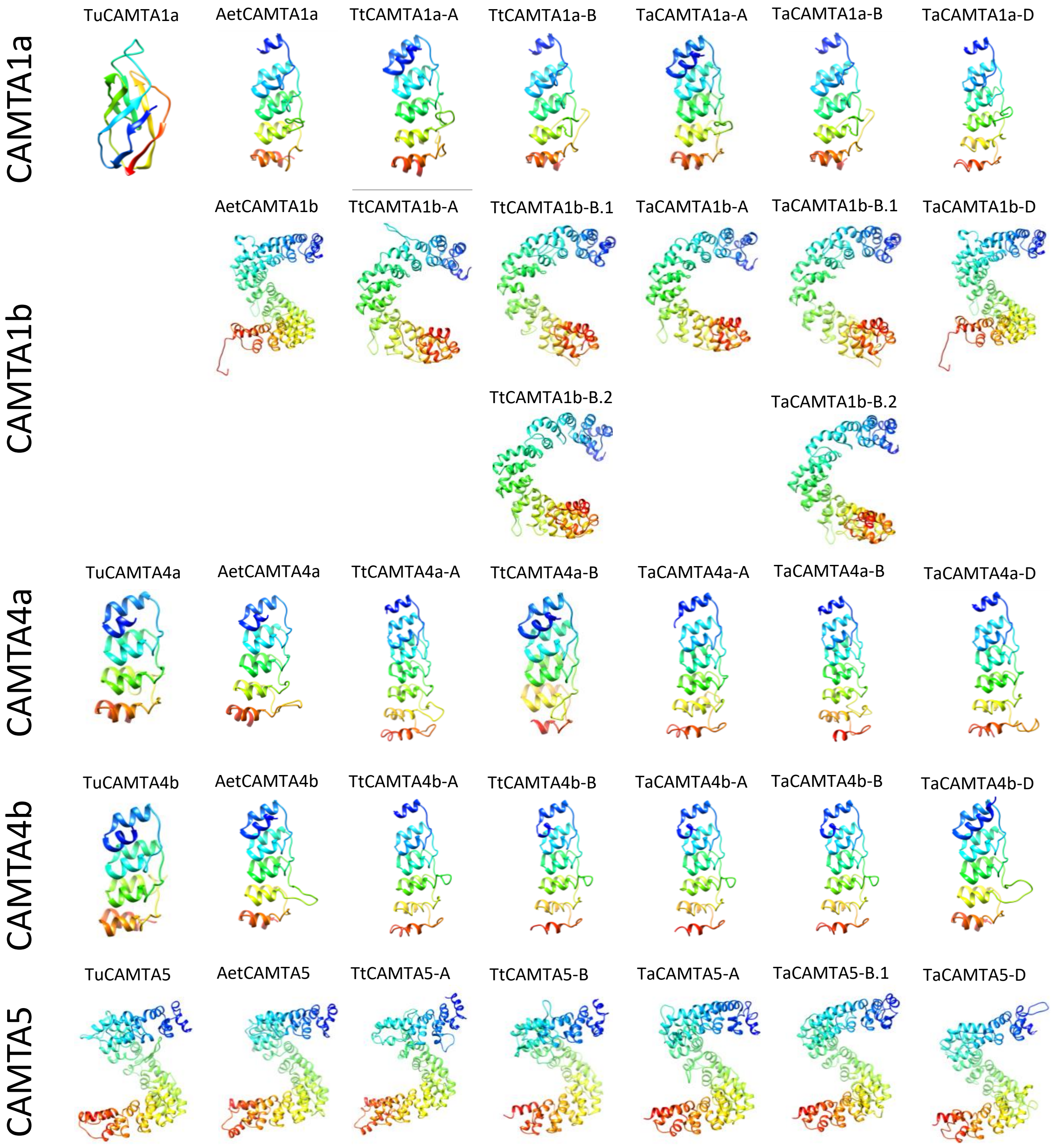

Figure S1. The predicted three-dimensional structures of CAMTAs family in *T. urartu* (AA)、*Ae. tauschii* (DD)、*T. turgidum* (AABB) and *T. aestivum*. The α-helixes and β-sheets are sequentially highlighted in different colors. Line is the random coil structure.

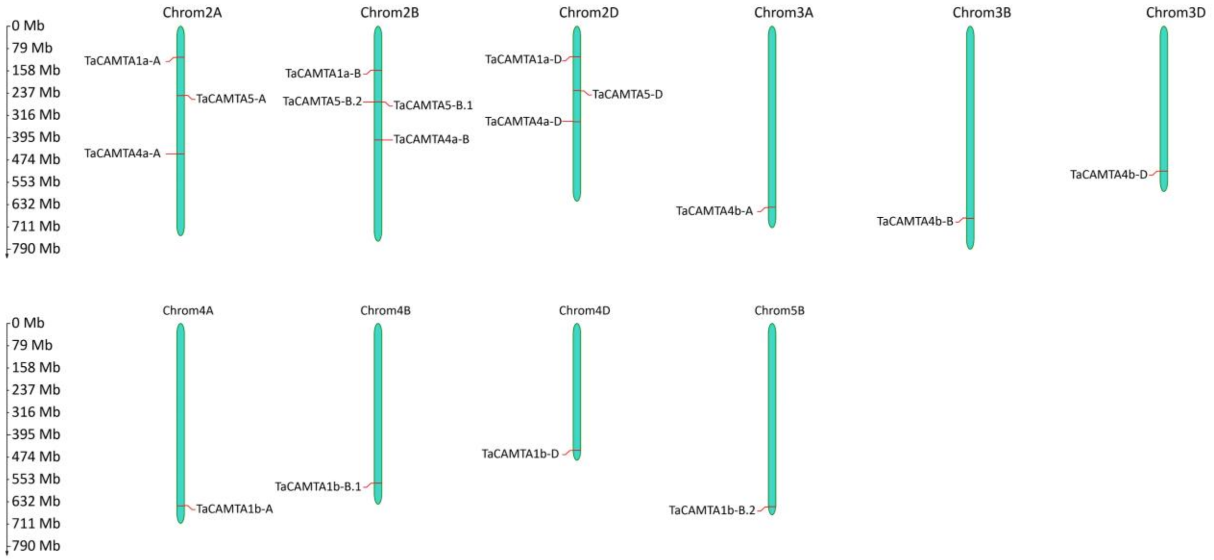

Figure S2. The chromosomal location of all *TaCAMTA* genes

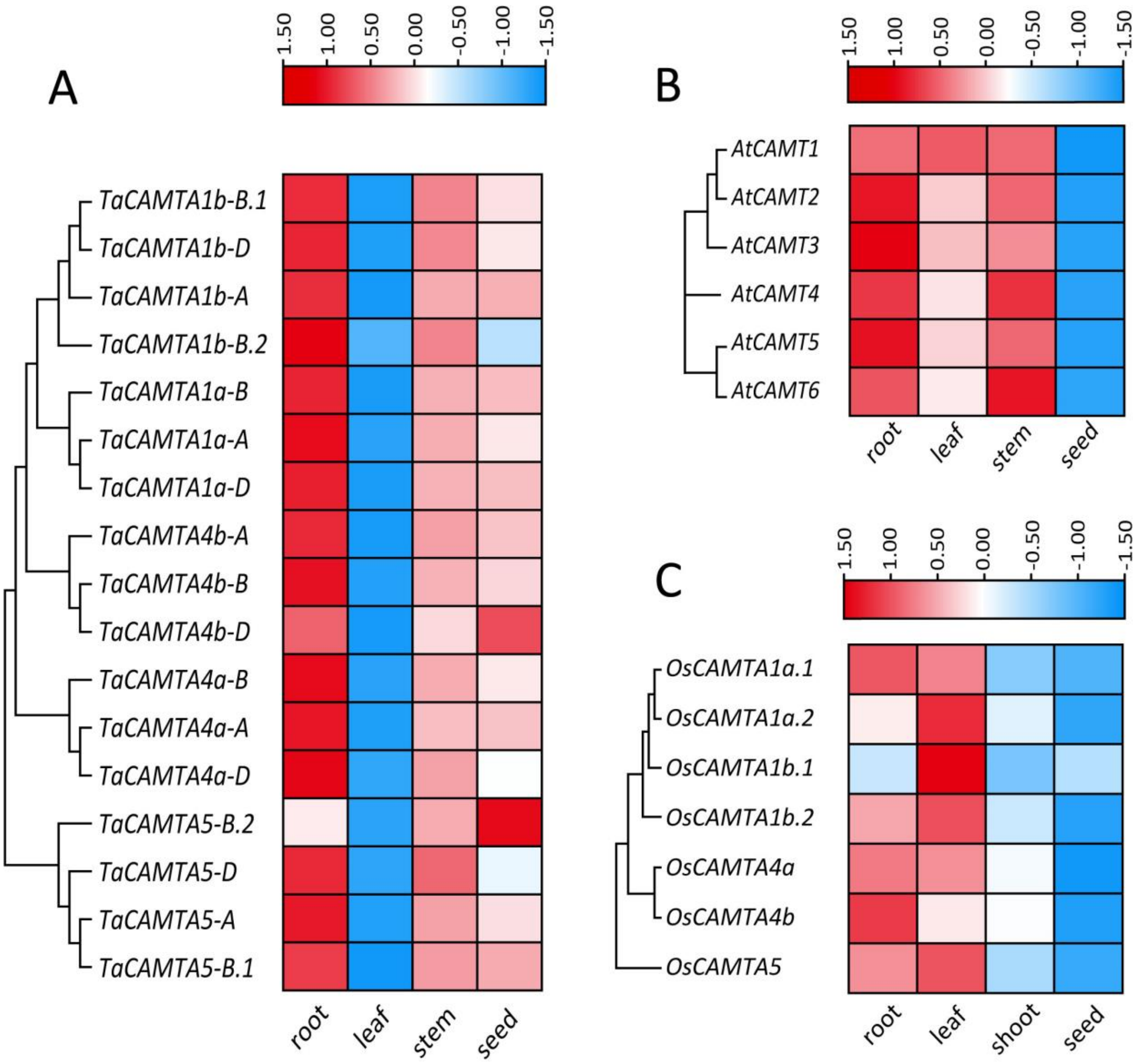

Figure S3 A-C. Heat map of expression profiles for wheat, Arabidopsis and rice CAMTA genes across different organs of seedling stages with the phylogenetic tree of them. The relative expression level of a particular gene in each row was normalized against the mean value by $\log_2$ transformation. The color scale below represents expression values, blue indicating low levels and red indicating high levels of transcript abundance.

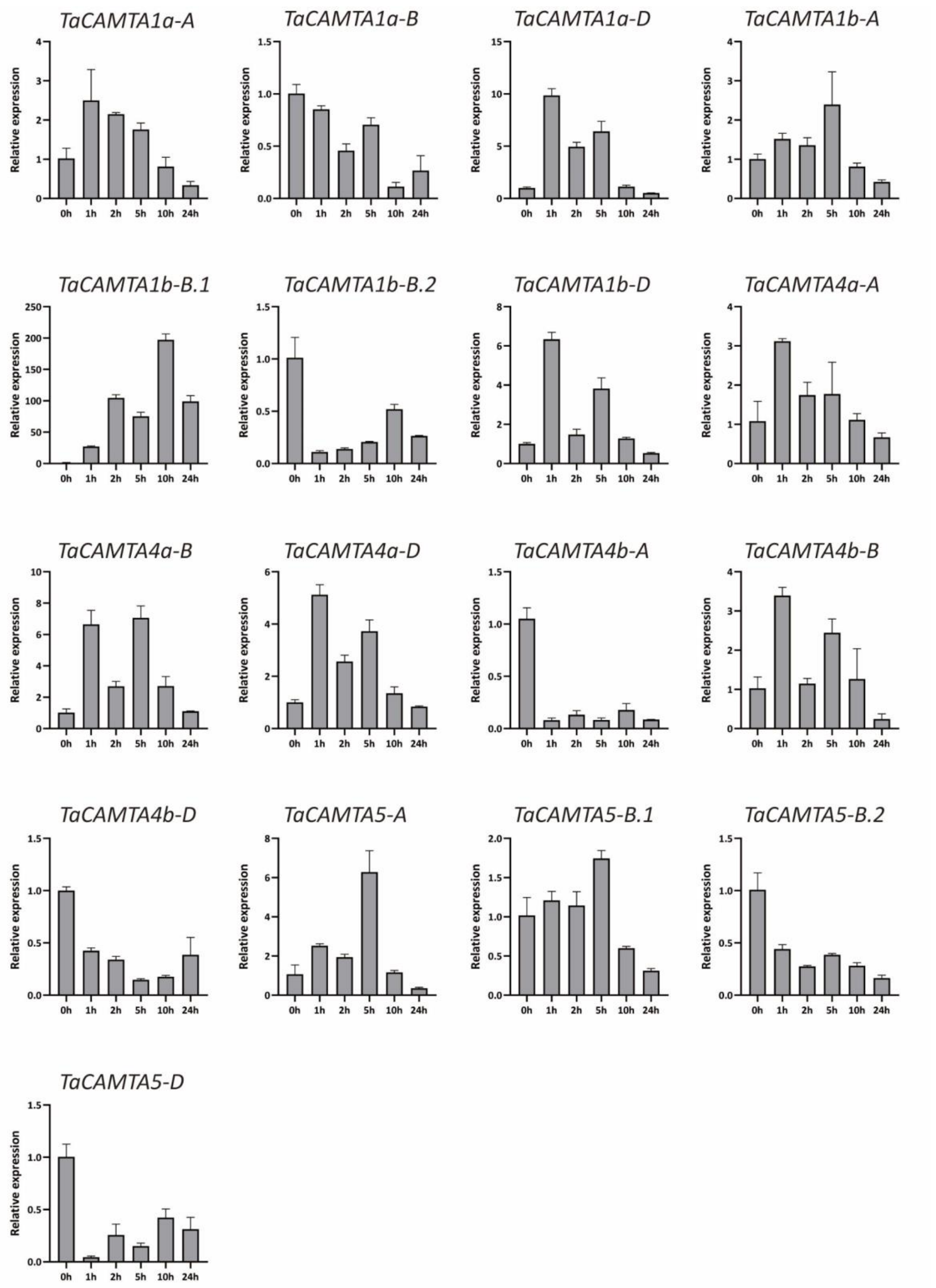

Figure S4. Expression analysis of *TaCAMTA* genes in the seedling stage of J18 under drought stress. The x-axis represents time after the onset of stress treatments. Error bars represent the standard deviations of three biological replicates.

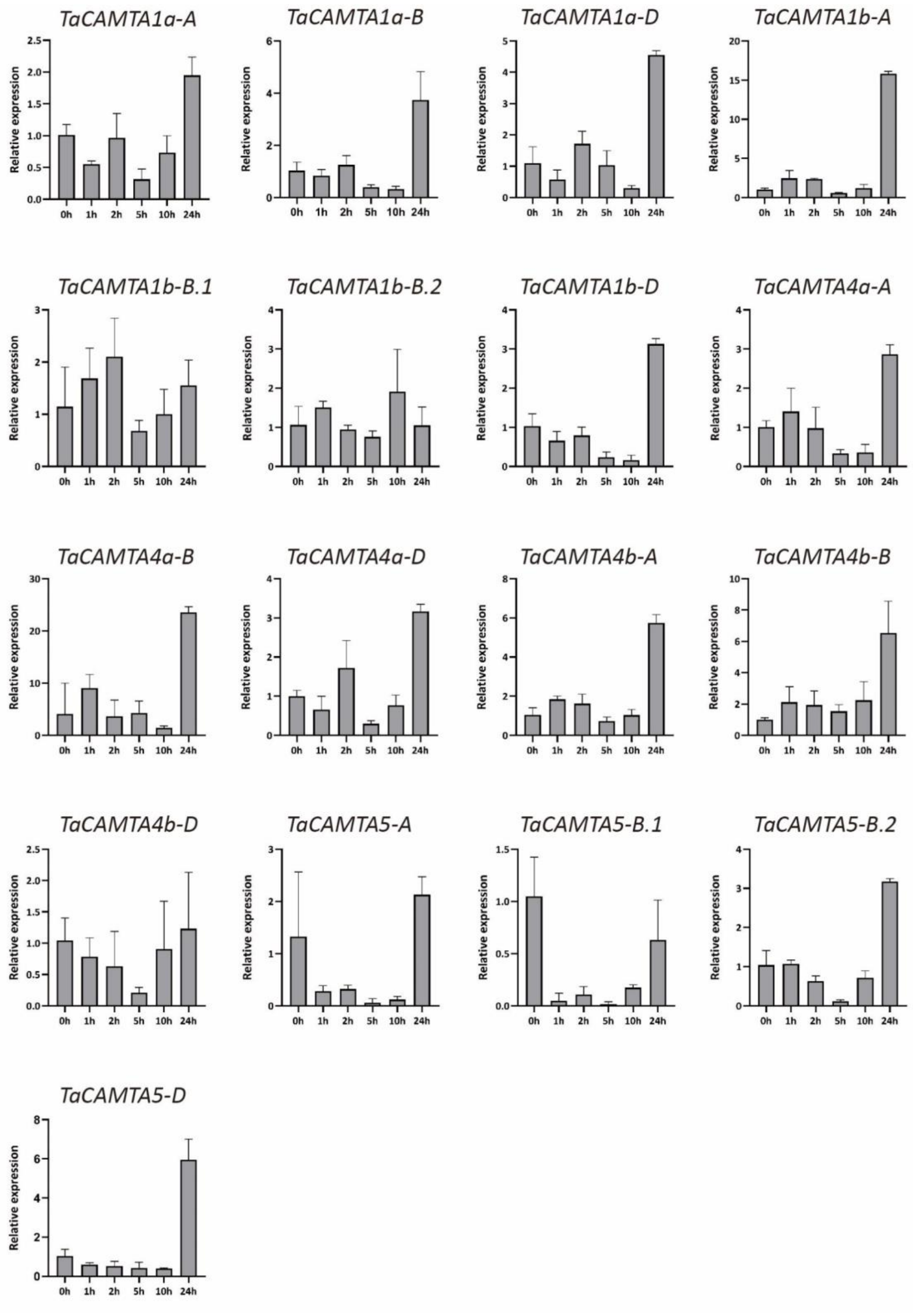

Figure S5. Expression analysis of *TaCAMTA* genes in the seedling stage of J18 under salt （200 mM NaCl） stress. The x-axis represents time after the onset of stress treatments. Error bars represent the standard deviations of three biological replicates.

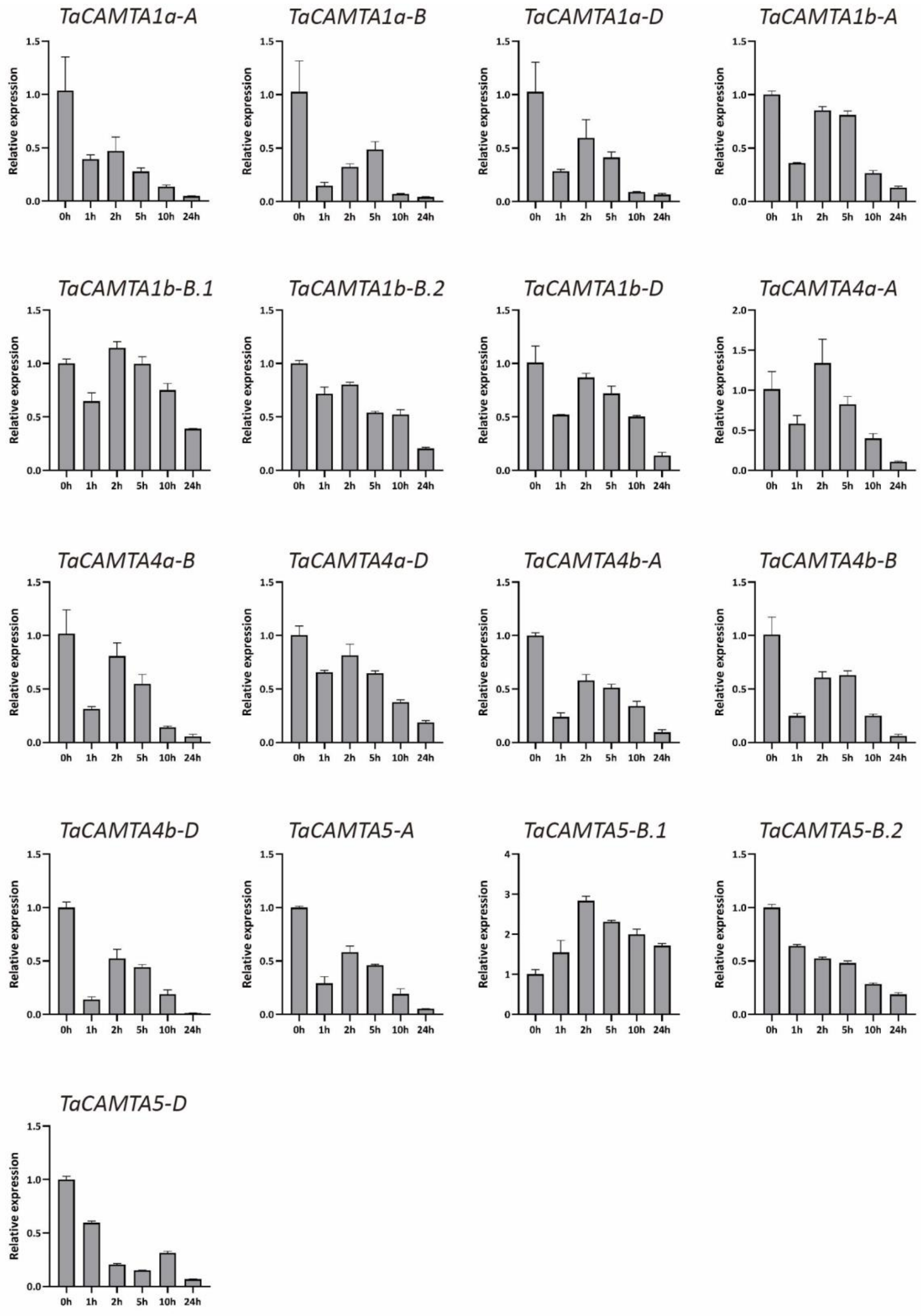

Figure S6. Expression analysis of *TaCAMTA* genes in the seedling stage of J18 under cold （4℃） stress. The x-axis represents time after the onset of stress treatments. Error bars represent the standard deviations of three biological replicates.

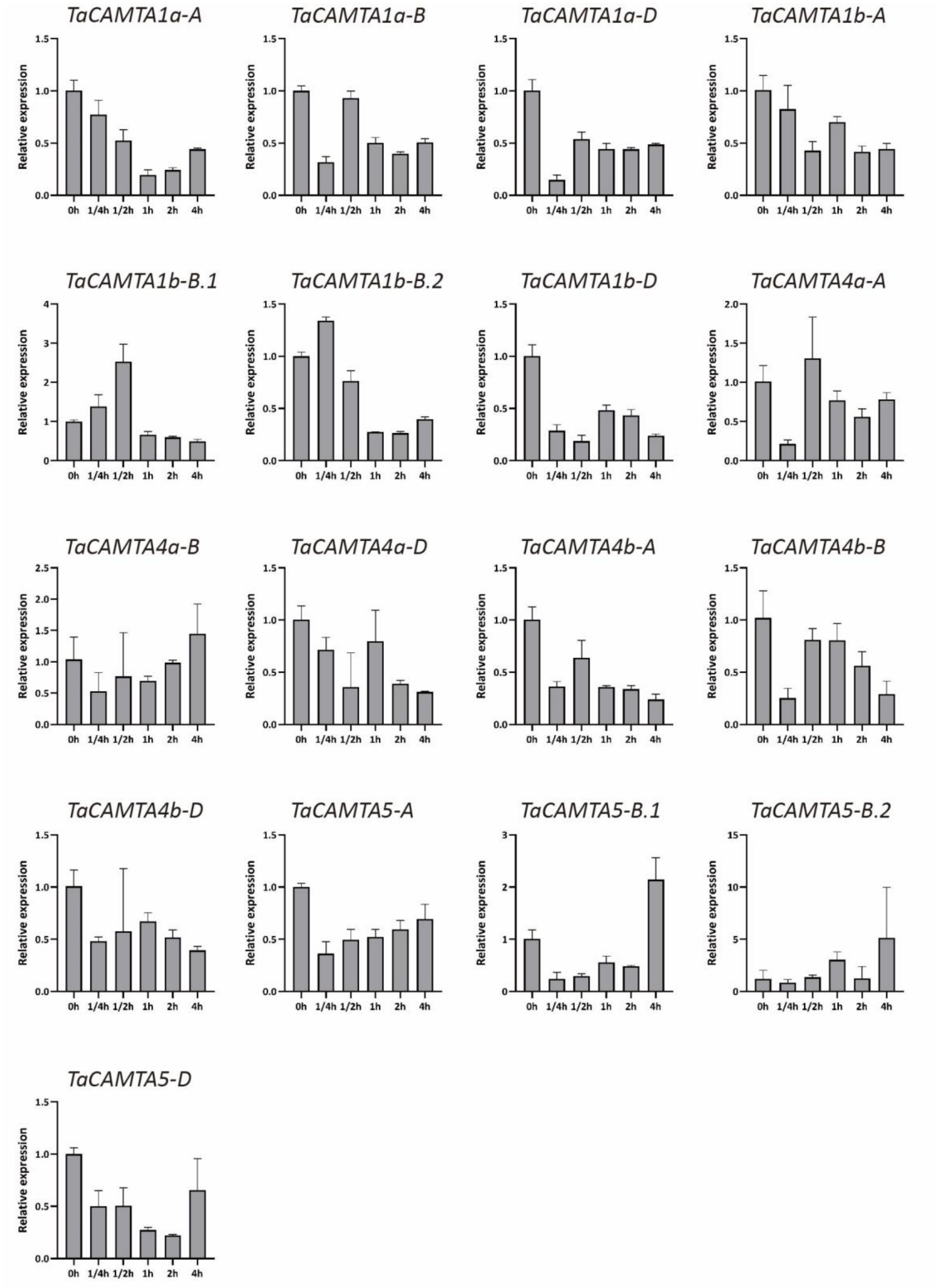

Figure S7. Expression analysis of *TaCAMTA* genes in the seedling stage of J18 under heat （40℃） stress. The x-axis represents time after the onset of stress treatments. Error bars represent the standard deviations of three biological replicates.

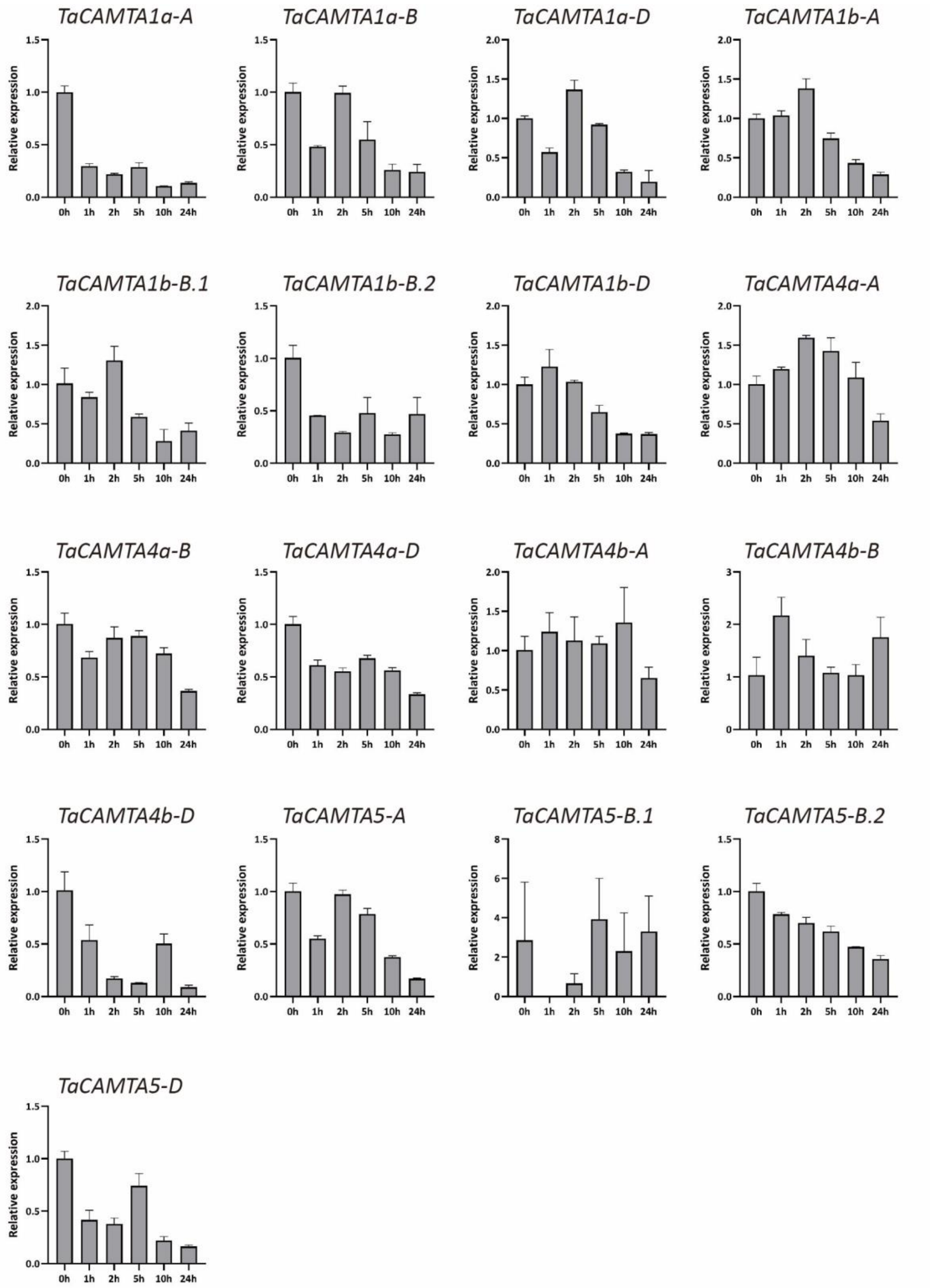

Figure S8. Expression analysis of *TaCAMTA* genes in the seedling stage of J18 under ABA （100 mM ABA） stress. The x-axis represents time after the onset of stress treatments. Error bars represent the standard deviations of three biological replicates.
